# A Qualitative Exploration of Paid Carers' Experience of Caring for Men With Intellectual and/or Developmental Disability and Harmful Sexual Behaviour

**DOI:** 10.1111/jar.70274

**Published:** 2026-06-30

**Authors:** Eleanor Drew, Lisa Richardson, Glynis Murphy, John Rose

**Affiliations:** ^1^ School of Psychology The University of Birmingham Birmingham UK; ^2^ Tizard Centre The University of Kent Canterbury UK; ^3^ Intellectual Disabilities Research Institute (IDRIS) The University of Birmingham Birmingham UK

## Abstract

**Background:**

Paid carers supporting men with Intellectual and Developmental Disability and Harmful Sexual Behaviour face complex challenges which can affect the carers well‐being and quality of care for the person they support. This study explores carers' lived experiences of caring for this group of men.

**Method:**

Six paid carers were interviewed regarding their experience of providing care. Semi‐structured interviews were conducted online and analysed using Interpretative Phenomenological Analysis.

**Results:**

Three superordinate themes were identified through the analysis: ‘The balance of drive and burden’, ‘External influence on care provided’ and ‘Co‐producing care and support’. Carers found the role emotionally demanding and needed support from others to cope. The importance of person‐centred care and carer responsiveness was also emphasised.

**Conclusion:**

This study highlights the emotional demands and negative public perception faced by paid carers. Clinical implications include enhancing professional recognition, strengthening organisational support and promoting emotional processing.

## Lay Summary

- This study explores the well‐being of direct care staff who care for men with Intellectual Disabilities who have exhibited Harmful Sexual Behaviour.
- Care staff were interviewed about their experiences and their interviews were summarised.
- Carers found the role emotionally demanding and needed support from others to cope.
- The results highlight the need to provide organisational support to carers to provide effective support to the people they serve.

## 1 Introduction

Paid carers play a key role in supporting men with Intellectual and Developmental Disability (IDD) and Harmful Sexual Behaviour (HSB), particularly in managing risk and influencing treatment outcomes (Svae et al. 2022). However, many carers report feeling ill‐equipped and lacking confidence (Saxe and Flanagan 2016). The importance of this caring relationship is also demonstrated by the potential beneficial outcomes, including behavioural change being linked to increased carer empathy (Sandhu et al. 2012). It is crucial that experiences of caring for this group of men are understood to ensure effective care is provided.

Paid carers can face several emotional and practical challenges, with a major challenge being a lack of organisational support. Carers have reported experiencing burnout linked to low reciprocity for their efforts from organisations (Thomas and Rose 2010). This can contribute to staff feeling vulnerable to compassion fatigue (Rose and Walker 2018). Research by Cope (2018) and Storey et al. (2011) highlights feelings of emotional isolation among carers, often feeling that they have limited opportunities to share their feelings at work, devaluing their emotional experiences. This can lead to carers feeling the need to suppress their emotions (Storey et al. 2011).

A second challenge for paid carers is stigma from the public, who may display negative and poorly informed perspectives of the work (Pelleboer‐Gunnink et al. 2021). Whilst some carers try to change community attitudes (Rose and Walker 2018), others are pushed towards social withdrawal (Mitter et al. 2018) and experience a diminished professional identity. Negative societal views can also impact the caring relationship, potentially compromising quality of care (Esmail et al. 2010).

The nature of the caring role can lead to increased burnout (Mills and Rose 2011), and in some cases vicarious trauma (Rose and Walker 2018). Exposure to distressing information, as well as contextual factors such as length of experience and care setting, can worsen this impact (Rose and Walker 2018).

### 1.1 Research Rationale and Aims

Despite growing awareness of the challenges faced by this staff group, research focusing on the experiences of paid carers remains limited and requires further exploration (Rose and Walker 2018). While NICE guidelines highlight the importance of providing training and emotional support to carers (NICE 2015), there is no specific guidance for carers working with HSB. Furthermore, most existing studies focus on experiences of Challenging Behaviour, rather than HSB. Organisational challenges relating to a lack of training and supervision are known to impact paid carers and their relationship with individuals (Robinson et al. 2023). However, HSB presents distinct challenges, including moral conflict and increased responsibility (Cope 2018). It remains unclear whether support strategies developed for managing Challenging Behaviour will transfer to staff working with HSB. Research exploring the negative impact of community attitudes (Ali et al. 2012) has focused on family members, overlooking paid carers. Exploring how paid carers experience community attitudes could allow organisations to tailor interventions to provide more effective support. Evidence suggests that if an organisation can provide appropriate support for staff, it can lead to safer and more effective care for the individuals they support (Rose and Walker 2018). Additionally, effective support for carers may serve to enhance retention and recruitment (Murray et al. 2022).

### 1.2 Aims

The present study aims to understand the lived experiences of paid carers supporting men with IDDs and HSB. It is hoped that gaining a deeper understanding of how carers perceive and navigate the challenges associated with their role will contribute to a broader understanding of this area. Additionally, the research aims to consider the emotional dimensions of the caregiving role.

## 2 Methodology

### 2.1 Context

This study was nested under a national Randomised Control Trial (RCT), the HaSB‐IDD trial, studying the effectiveness of a Cognitive Behavioural Therapy (CBT) intervention for men with IDDs and HSB, including carers' perspectives. The current study sampled carers who were recruited to the RCT and were not involved in the specific treatment.

### 2.2 Design

#### 2.2.1 Ethics

Ethical approval for this study was obtained through the HaSB‐IDD trial and granted by the NHS Health Research Authority. Participants were provided with information sheets detailing the purpose of the study and what would be asked of them if they participated. If the participants verbally agreed to be interviewed, they were asked to complete a study consent form, providing written consent.

#### 2.2.2 Recruitment

Paid carers were the group of interest in this study. The inclusion and exclusion criteria (Table 1) were developed using the RCT study protocol. Ensuring all participants met these criteria ensured homogeneity within the sample, an important feature of small samples in IPA studies (Smith et al. 2022).

**TABLE 1:** Participant eligibility criteria.

Inclusion criteria	Exclusion criteria
Paid carers	Family members, informal, unpaid carers
Caring for a man over 18 years old, with Intellectual and Developmental Disabilities and Harmful Sexual Behaviour	
Harmful Sexual Behaviour defined as illegal sexual behaviour against a person who was not consenting. Not necessarily reported to or prosecuted by the police. Occurred within the last 5 years	
Caring for men in community or inpatient healthcare settings (low‐medium secure)	Caring for men in prison, probation or high secure services
Participating in the inactive arm of the clinical trial	Participation in the active arm of the clinical trial

The process for recruitment started with identifying suitable participants from the trial who were caring for participants in the control arm. Out of a possible 14 participants, six of these consented to be interviewed.

#### 2.2.3 Participants

The research was conducted across the United Kingdom. The names and details of the participants were changed to protect their anonymity, and location details were withheld. Pseudonyms and details of participant roles are shown in Table 2.

**TABLE 2:** Participant demographics.

Participant code	Gender	Age	Ethnicity	Job role	Caring experience	Employer
Gemma	Female	30–39	White British	Therapy Technician	2 years	NHS, residential
George	Male	50–59	Black African	Community carer	17 years	Private, community
Jenny	Female	30–39	White British	Deputy Manager	> 19 years	Third sector, community
Liz	Female	50–59	White Scottish	Senior Care Support Worker	27 years	Public sector, community
Simon	Male	21–29	White British	Registered Manager	11 years	Private, residential
Alan	Male	> 60	White British	Clinical Support Worker	3 years	NHS, community

Each carer supported a man identified as having an Intellectual and/or Developmental Disability and a history of HSB. Whilst the clinical cut off for a diagnosis of Intellectual Disability is often cited as IQ below 70 (BPS 2015), individuals with a higher IQ were included in this study to reflect a more clinically meaningful definition. The BPS (2015) have recognised that using a cut off can be an arbitrary figure that may not always represent an individual's level of functioning. Similarly, Boat and Wu (2015) highlight the DSM‐5 diagnostic framework's choice to remove the numeric IQ threshold when defining Intellectual Disability, recognising that it should be assessed by impairments in social and adaptive functioning, rather than a fixed IQ score. It is important to note that although one man had a FSIQ of 82, the trial managers used clinical judgement to determine their suitability for inclusion.

The individuals whom the participants supported were all men, five of whom lived in community facilities and one in an inpatient residential facility. Their IQ was measured by the WAIS and ranged from 51 to 82 (Mean 61) and their ABAS scores from 49 to 76 (mean 59). Their offences included a range of contact and noncontact sexual offences. Contact offences included, inappropriate touch, non‐penetrative sexual acts with minors and allegations of rape. Non‐contact offences included unwanted text messages and voyeurism.

### 2.3 Data Collection

A semi‐structured interview schedule was used during interviews. Each interview was audio recorded. Interviews were conducted between May 2024 and January 2025 and lasted between 1 h and 1 h and 15 min; the audio recordings were transcribed verbatim. An overview of the topic areas covered in the interview is shown in Table 3.

**TABLE 3:** General topic areas covered by the interview.

Participant Information Relationship to the client Experience of supporting client Perception of others/community acceptance Exploration of therapeutic engagement Access to support

### 2.4 Data Analysis

Transcripts of the interviews were analysed using Interpretative Phenomenological Analysis (IPA) (Smith et al. 2022). Because the research was directed towards the lived experiences of paid carers, it was felt that IPA was an appropriate methodology.

This analysis resulted in three Group Experiential Themes and six subthemes being identified across the participants, providing understanding of the meaning they make of their experiences of caring.

### 2.5 Reflexivity

Due to the nuanced and subjective nature of qualitative research, an important component is reflexivity (Olmos‐Vega et al. 2023), which can be defined as the researcher's evaluation of themselves and their impact on the findings (Shaw 2010). Reflexivity was an ongoing and embedded process in the research, acknowledging the understanding and context the researcher is bringing to the analysis.

This is an important aspect of qualitative research as both researcher and participants are humans interacting with the world (Shaw 2010), and therefore, can have an impact on analytic interpretations of the data. Within IPA, double hermeneutics means that much of the analysis is based on interpretations made about participants' sense‐making (Eatough and Smith 2017). The primary researcher therefore took time to reflect on their professional experience (Braund et al. 2024) of working within an adult community learning disability service and how this had been a motivating factor in studying this phenomenon. Thus, understanding that the researcher and their experiences can impact the interpretation of the data.

Following guidance from Levitt et al. (2021), techniques were used throughout the study process to ensure interpretations were grounded in the data and that they remained open to alternative perspectives. Research supervision was used regularly throughout the analysis stage where emerging concepts were discussed. A reflective diary was kept to document when interpretations emerged and any preconceptions were made. Initial thoughts during each analytic stage were documented separately and referred to when generating the PETs. These notes were reviewed after the PETS were grouped, allowing the primary researcher to maintain an open mind.

Once initial group experiential themes had been drawn from the data, the primary researcher attended a carers group meeting to share the preliminary findings. This participatory method, as recommended by Levitt et al. (2021), provided an opportunity for the experts by experience to share their thoughts on the themes and enhanced the ability to reflect on the development of the experiential themes. This ensured they were grounded in the data, while also allowing the ability to explore alternative perspectives (Barrett et al. 2020). This provided an opportunity to understand if the themes fit with the carers' own experience and what this could mean for future developments.

## 3 Results

**FIGURE 1:**
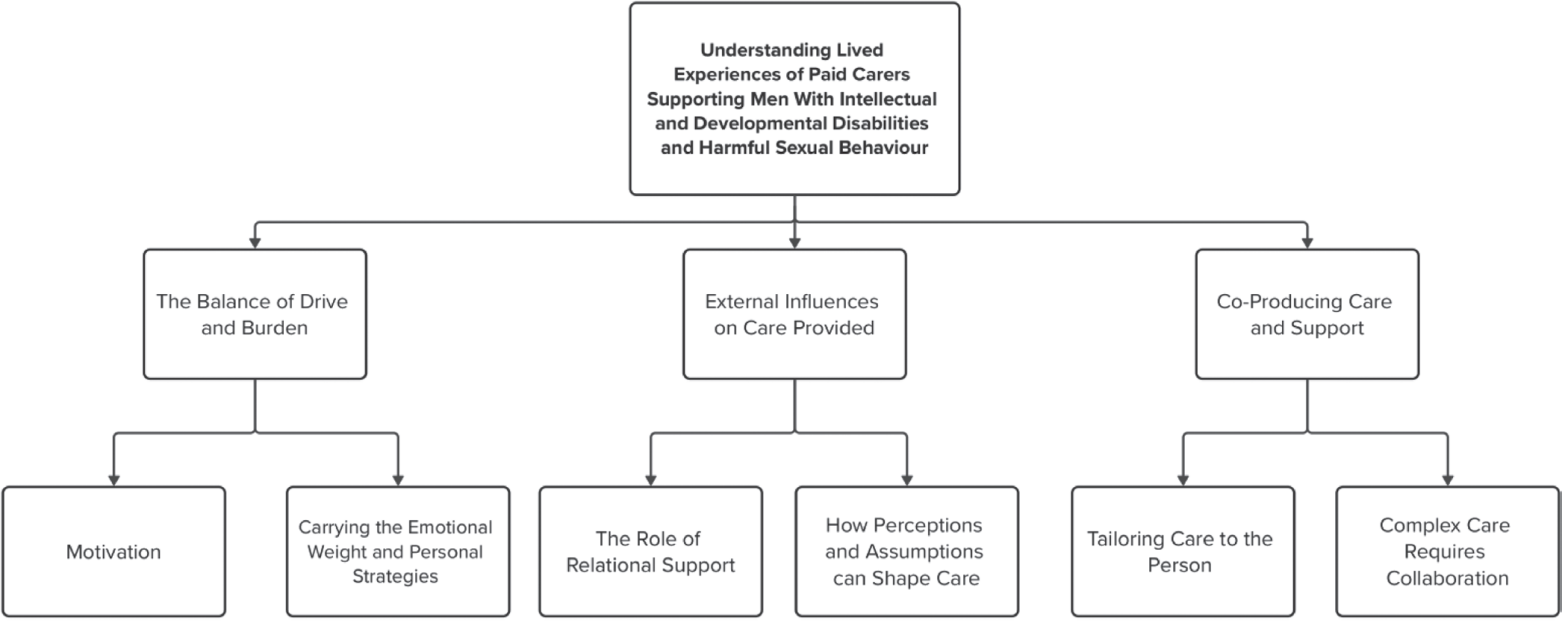
A thematic map of group experiential themes and subthemes emerging from the data.

Three key themes emerged from the analysis of the data: ‘The balance of drive and burden’, ‘External influences on care provided’ and ‘Co‐producing care and support’. Each theme had two subthemes, displayed in Figure 1.

### 3.1 Theme 1: The Balance of Drive and Burden

Theme 1 describes what is required from carers to be able to care for people. This includes the personal values and principles that the carers hold, as well as strategies they develop that allow them to navigate the complex demands of the job. They describe how accepting what cannot be changed and understanding the limits of their role were central to this adaptation. Additionally, carers describe their reasons for choosing this work and the motivation that keeps them in the role.

#### 3.1.1 Subtheme 1.1: Motivation

Because of the challenges of the job, carers must be intrinsically motivated to do the role, and many describe this as central to their practice. Participants who care for men with both contact and non‐contact HSB reported noticing positive changes in the men they work with, being able to educate others and feeling appreciated in their role as intrinsically motivating factors.

For some participants, being able to improve the lives of the men was a key motivation for doing the job, “So it's just like giving people more independence to try new things and to move on with their lives and to be positive and bring positivity to their lives” (George). Providing this sense of independence for clients may be even more pertinent for men who have faced restrictions due to their risk behaviours.

Just shows me exactly why I have to do my job and advocate for people with a learning disability you know it just goes to show that's what we do for a reason and we need to keep doing that and banging that drum about learning disabilities for people to know (Jenny).

For others it was the relationship they had built with the men that allowed them to tolerate the emotional challenges of the role. Building a strong relationship allowed carers to take enjoyment from the work including shared activities, particularly when these were novel to the man they support, “I have taken him on weekends away…tours away for the weekend, which is a positive he really enjoyed that. That's something that he's not done since being in foster care as a child” (*Simon*). In Simon's case enjoyment appears to come from providing opportunities which could be seen as healing, having not experienced such events since childhood.

The carers also described the motivation gained from observing changes in the men and the impact of their work. These changes felt even more valuable when their work was noticed and appreciated by others, “Yeah some people will say, ‘oh you are doing a wonderful job’ and they appreciate it, they appreciate what we do” (*George*).

Despite the complexity of the work, all the carers remained clear on their reasons for doing the role. The depth of the carers' motivation appears closely linked to the context of the men's lives and their presentation of HSB.

#### 3.1.2 Subtheme 1.2: Carrying the Emotional Weight and Personal Strategies

All participants described experiencing the job as emotionally heavy at times, “And he's quite draining because he will go on and on and on at you and he doesn't talk to you he talks at you” (*Jenny*), and that it had been necessary to develop personal skills to manage this. Carers described being overwhelmed by the communication from the person they support and the knowledge they were expected to hold in their mind to be able to support them, “Yeah I think it's more emotionally and mentally draining sometimes because of all that thought process that has to go into it” (*Gemma*). Carers described competing demands to provide emotional support and manage risk. One participant described how they felt this responsibility fell on them.

At times it was taxing at times, quite overwhelming some days you know. Trying to think, I wouldn't say it was stressful I would say it was more, a lot of the onus on me, a lot a lot (Alan).

One way carers managed these demands was being clear on the remits of their role. Some described developing tolerance to accept what they cannot change, “This is how he chooses to live, we have to accept that (Jenny)”. This description refers to the carer's acceptance of one man's living standards so as not to compromise their relationship, rather than demonstrating passivity to providing support. Whilst others described choosing to go above and beyond normal expectations for the good of the men. In some cases, this meant doing something that their organisation would not have required.

You go out, you go over, above and beyond, to do things that you're not really supposed to do, but the positives far outweigh the negatives, do you know what I mean? If that means he's in a good mood, that means he's stable, that means he's not offending, I'll do it (Alan).

When talking about the impact of the man's past HSB on their role as carers, participants described needing to detach themselves from the emotional impact of this, “At the end of the day you have to detach the crime from the person” (Jenny). This detachment was described in the context of separating work from home and the individual from their behaviour.

Acknowledging the challenges of their role and being able to implement strategies to protect themselves is described as vital for being able to provide care for men with IDDs and HSB. In relation to managing the strain of the role, the strategies implemented were directed by the carer rather than their organisation. Thus, indicating that across public and private sector organisations the carer bears the weight of protecting their own emotional well‐being.

### 3.2 Theme 2: External Influences on Care Provided

This theme builds on the emotional challenges of the role explored in Theme 1 and highlights the added complexity that the work doesn't occur in isolation. Carers are required to negotiate the tension between working directly with the man, as well as navigating the impact of other people. All participants highlighted that their work can be heavily influenced by people outside their relationships with the men they support.

#### 3.2.1 Subtheme 2.1: The Role of Relational Support

Carers described that their job role was heavily influenced through receiving support from other people, namely other professionals. Carers discussed needing to be open to this support and accept when they may need to turn to others to look after themselves, to be able to continue caring. An important point raised was that personal life can have an impact on a carer's ability to perform the role too. Being able to raise personal concerns in supervision was something the carers found helpful. The workplace setting is an important context when considering this, as organisations can be responsible for creating a culture in which supportive discussions are enabled, as described below by Alan who works in the community.

Because there might be something going on in the background, you might have issues going on at home that you're not telling anybody about. So, in supervision, as much as you talk about some patients stressing you out, but you can also say, ‘well actually I've got this going on as well’ (Alan).

Participants found this support from management most helpful when it was readily available, something especially important for carers who are lone working with the men.

Importantly, it was highlighted by several participants that support was equally beneficial when it was from peers, rather than management, “I've got the support of the team. We just work well together, I think we can all cope with any challenges [the man] brings because we're quite tight” (*Liz*). This reflects peer empathy based upon shared caring experiences.

As well as being able to share and offload challenges of the role, carers reported that support from others allowed them to problem‐solve and develop a new perspective on the person they supported, “then we will discuss and come up with a solution” (*George*). Similarly, participants described the benefit of receiving training from others and working jointly with different professionals to provide effective care and support. This collaboration was viewed as broadly positive and enhanced the effectiveness of their care.

#### 3.2.2 Subtheme 2.2: How Perceptions and Assumptions Can Shape Care

The carers' role is also influenced by individuals outside the caring profession, namely the public. Carers commonly noted that members of the public made broadly negative assumptions about the people they were supporting and the work the carers were doing, this seemed to be based on minimal understanding, “’Ooh bad, horrible, paedophiles', and there is no understanding of what might have led them to that and what challenges they have faced in life. I would say there is none” (*Liz*). Liz's consideration of the public narrative appears to capture the binary view of individuals as either good or bad. However, her reference to understanding that this can be morally ambiguous and her role in caring causes her to acknowledge this.

Participants felt that communities were unable to see past offending behaviour which led to stigmatising attitudes, “There is still that stigma from society, I think there always will be” (*Jenny*). Carers felt that this stigma surrounding offending behaviour could have been partly the result of a lack of knowledge about Intellectual Disabilities which led the public to make assumptions about the men. There were occasions in which this negative perception had a direct impact on the carer's job role. In one case, the carers changed their contact with a man they supported.

So, when that happened because of the vigilantes… we had to reduce, we had to look at the risk, reduce our contact, and when we saw him we had to be in twos and most of the time we saw him at the hospital, to be honest (Alan).

However, there had been occasions in which the lack of awareness of the man's difficulties had led to surprisingly positive interactions with members of the public, “They get along very well. I think potentially, if there was an awareness that they had sexualised background that could potentially change perceptions, but at the minute I think everyone's alright” (*Simon*). This reflects the complex decision‐making carers face around transparency and protection.

### 3.3 Theme 3: Co‐Producing Care and Support

The final theme to emerge from the data was the commitment of carers to put the men's needs before their own. To do this, the carers needed to have a strong understanding of what the man wanted from the caring relationship, and they relied on them to openly communicate this.

#### 3.3.1 Subtheme 3.1: Complex Care Requires Collaboration

As these men present with complex support needs and risk histories, collaboration is a necessity for relational care and safety. Carers were required to build strong relationships with the men they supported, built on understanding and listening to their needs and views. This is especially vital given the potential for communication difficulties. However, ultimately, the strength of this relationship was determined by how much the men were willing to share with the carer.

It takes time for him to talk to you. You know, the more he knows the staff, the more he opens up and he talks, but when you start working with him, he is a bit laid back he doesn't mind, but once he gets to know you, he can talk to you (George).

Whilst a good understanding of the man strengthens the relationship and allows the carer to work effectively with them, many felt their role was limited by what the man was willing to do. For example, Alan described having to change the offer of support and how he worked with the man he was supporting because they continuously chose not to engage and were focused more on what they enjoyed doing, “I stopped offering (support appointments) because I know he won't engage with them. He just doesn't, he's more like a social person” (*Alan*). This demonstrates a carer being adaptable and enabling him some agency. Although the scope for flexibility in the community setting also led to challenges such as enforcing attendance, as described by Alan.

The main one was missing appointments; he was notorious for it. I mean because when he missed [the appointments] that meant I was having to move my appointments round, to make sure he was at the next one the following week (Alan).

In contrast to this, Simon described his work being impacted by the external restrictions placed on the mandate to the HSB and found this to be a motivating factor for engagement.

He understands it slightly. He knows that it means he has to be staffed, he knows it means that if he goes back to hospital, then the chances of him getting out again would be slim to none. So, I think that is a massive factor in him cooperating a little bit more with the conditions (Simon).

Finally, carers shared their experiences of having to advocate for themselves within the role and needing to work with the men to ensure they got some space, once the men's needs had been met. This appeared particularly relevant for staff working in community settings who provide 1:1 care.

I would just say to him, ‘I need a break; I'm going to the sleepover room for 30 minutes’. He likes to know exactly what time you're going to be back, and then he'll watch the telly or something. I'll say ‘when we come back we'll do that’ and he'll give me, he's got a lot better at giving me half an hour if I need it. He never used to, so we didn't ever get a break (Liz).

The experience from all carers can be seen to demonstrate that cooperation and collaboration with the client, whatever it is motivated by, is an important factor for effective co‐production of care and support.

#### 3.3.2 Subtheme 3.2: Tailoring Care to the Person

Carers highlighted that they were required to have a clear understanding of what worked for the man they supported, which had developed through long‐term placements and a number of years of providing support to them.

He needs to talk things through; he likes to talk things through constantly. I think that he just needs you to sit and listen to him even. You don't even need to say, don't need to say much, just need to sit near him and listen (Liz).

Two carers described that the way they would respond to the men was dependent on how they were communicating with them; they were led by the men's presentation, demonstrating the need to employ professional judgement. Carers were required to decide what was going to be most beneficial in the moment whilst creating boundaries: “I'm very firm but fair with him, I have clear boundaries with him” (*Jenny*), an important skill when working with men who display HSB.

## 4 Discussion

The theme ‘Balance of drive and burden’ demonstrates the personal development carers undergo to manage the emotional weight of the job. Carers described the need to set clear boundaries on what they were prepared to do to meet the needs of the men and to emotionally detach from their work. This aligns with previous research by Cope (2018) and Storey et al. (2011), who found that detachment related to a lack of emotional outlets for staff, similar to the findings in the current study. Excessive detachment can risk reducing empathy in carers (Lee and Kiemle 2015), and therefore, highlights the importance of supervision and emotional support for this staff group. This important finding reveals an experience unique to paid carers, as family caregivers are likely unable to use this strategy of compartmentalising (Perera and Standen 2014).

The second important facilitator for doing the job was motivation. Carers described a deep commitment to improving the lives of men they support, which was validated by their work being appreciated by others. This supports the findings from Stevens et al. (2021) that staff need to feel they are improving the lives of people they support. Understanding what motivates carers to do the role is important because support work is often underappreciated and underpaid (Hastings 2010), both of which have been linked to burnout in healthcare professionals (Renger et al. 2020). There is a case to be made for formalising the role through clearer ethical guidelines and professional standards, enhancing this recognition from others and enabling people to take greater pride in their work. There is also evidence to suggest that developing training requirements and higher pay may lead to improved retention (Murray et al. 2022). This is important to consider in the UK, where turnover in the adult learning disabilities workforce was over 29% in 2018 (Skills for Care 2018).

The ‘External influences on care provided’ theme indicates how external factors can shape a carers' experience. Relational support from colleagues is important for carer well‐being, echoing previous findings that peer support is an important coping mechanism (Rose and Walker 2018). Participants emphasised that this support needs to be frequent and accessible. Peer support was seen as automatic from colleagues and equally effective for promoting well‐being as supervision from management. Thus, suggesting that organisations should actively create spaces for peer connection and informal debriefs. Participants also strongly valued the availability of training and felt this supported effectiveness in the role.

Participants reported feeling negatively impacted by the public's lack of knowledge leading to misunderstanding and stigma surrounding the men they support and their work. Current participants described how stigma from the community created barriers to care delivery, supporting findings by Rose and Walker (2018) that negative societal attitudes can increase the difficulty of the caring role. It highlights the need for organisations to provide training to staff in addressing this stigma as well as understanding the impact of this on people with IDDs (Pelleboer‐Gunnink et al. 2021) and empowering them to have ways to resist stigma (Scior et al. 2022).

It is also important to recognise that carers' experiences are shaped by the wider context in which they work. For example, supporting men in the community can provide greater opportunities for rehabilitation and community integration but may also present higher levels of risk.

The final theme, ‘Co‐producing care and support’ reflected the carers' prioritisation of the men's needs above their own. This required highly individualised care, strong communication and collaborative relationships to co‐produce care and support. Participants stressed the importance of tailoring support to what men want and are willing to engage in, consistent with Svae et al. (2022) who found that effective care is difficult if people are unwilling to engage or accept it. These findings are consistent with theories of offending described by Keeling et al. (2009) which propose that difficulties with social and affective functioning can contribute to offending.

All of these issues can influence the relationship and how staff interact with the person they support. Organisational and staff factors, such as inpatient or community services, also have an impact. Inpatient settings may offer greater access to supervision, stricter routines and a controlled environment, whereas community settings may involve greater autonomy, but also exposure to the public who are likely to have a poor understanding of the situation. These contextual differences are important to recognise because they can influence paid carers' ability to build trust and maintain therapeutic relationships with clients with IDD and HSB. They constitute important factors in practice and policy in commissioning services, in that a range of services are required to meet the individually assessed needs of the people concerned. While community services are likely to provide the best opportunities for support and rehabilitation, they do need to be appropriately structured and resourced.

### 4.1 Clinical Implications

#### 4.1.1 Professional Recognition

Formal recognition of carers as a professional group could positively impact external perceptions of the role, their own sense of value as well as the effectiveness and quality of care. This recognition could foster a stronger professional identity for carers, particularly when paired with structured training programmes, enhancing carers' current strengths and capabilities. In the last 15 years, the number of learning disability nurses in the NHS has reduced by 43% in the UK (Royal College of Nursing 2023), suggesting the need to reinvigorate this, and similar training, for staff working in this demanding area.

Furthermore, training focused on enhancing a theoretical understanding of HSB could better equip carers to manage complex needs (Keeling et al. 2009) and facilitate the implementation of strategies men themselves may have learnt through individual or group treatment programmes.

#### 4.1.2 Organisational Support

Organisations play a key role in supporting carers' hard work and recognising contributions. Appreciation emerged as a key motivator in the current study. Organisations could enhance this sense of value by adopting a strengths‐based approach that values adaptive working styles and personalised care, serving to enhance the well‐being of carers. This includes seeking to enhance opportunities for carer contributions to be recognised through relationship‐building initiatives (Hamilton et al. 2024) or financial incentives (Stevens et al. 2021).

#### 4.1.3 Emotional Processing

While some emotional detachment from the role can be an effective coping strategy for carers, excessive distancing can hinder provision of care. Evidence suggests that secure attachments with carers are vital for the emotional well‐being of the people they serve (van Wingerden et al. 2018). Therefore, it is crucial that carers have access to safe and effective spaces to process their own emotional experiences and remain empathic to the people they support, such as reflective practice and supervision (Rose and Walker 2018).

This study echoes the importance of peer support and supervision in managing the emotional demands of the work. These findings align with guidance from Skills for Care (2018) and the Care Quality Commission (2013), both of which emphasise the importance of effective supervision for staff working with people with IDDs.

### 4.2 Strengths and Limitations

The range of participants, both male and female from various UK locations and employment sectors can be seen to enhance the representativeness of the findings, providing an understanding of the caring experience across different settings. The homogeneity of the sample (all paid carers) aligns with IPA methodology. However, this focus limits the generalisability and transferability of findings. The study was conducted within a larger clinical trial, which aided recruitment of participants but also presented challenges. Limited direct access to participants may have impacted recruitment and engagement. Furthermore, logistical constraints tied to the clinical trial occasionally complicated ethical procedures and timelines of the research. Finally, while the six‐person sample allowed for in‐depth analysis of the individual participant experience, it restricts the breadth of conclusions that can be drawn about carers' experiences more generally.

## Funding

This project/study is funded by the NIHR (NIHR/HTA project 128550). The views expressed are those of the authors and are not necessarily those of the NIHR or the Department of Health and Social Care.

## Data Availability

Research data are not shared.
